# Genetic fragmentation in India’s third longest river system, the Narmada

**DOI:** 10.1186/2193-1801-3-385

**Published:** 2014-07-28

**Authors:** Gulab D Khedkar, Rahul Jamdade, Amol Kalyankar, Anita Tiknaik, Tetsuzan Benny Ron, David Haymer

**Affiliations:** Paul Hebert Centre for DNA Barcoding and Biodiversity Studies, Dr. Babasaheb Ambedkar Marathwada University, Aurangabad, 431004 India; College of Tropical Agriculture and Human Resources, Department of Human Nutrition, Food and Animal Sciences, University of Hawaii, Honolulu, HI USA; Department of Cell and Molecular Biology, University of Hawaii, 1960 East-West Rd, Honolulu, HI 96822 USA

**Keywords:** The Narmada river, Genetic fragmentation, Falls, Dams, Fish, Dloop, Migration

## Abstract

India’s third longest river, the Narmada, is studied here for the potential effects on native fish populations of river fragmentation due to various barriers including dams and a waterfall. The species we studied include a cyprinid fish, *Catla catla,* and a mastacembelid, *Mastacembelus armatus*, both of which are found in the Narmada. Our goal was to use DNA sequence information from the D-loop region of the mitochondrial DNA to explore how this fragmentation could impact the genetic structure of these fish populations. Our results clearly show that these barriers can contribute to the fragmentation of the genetic structure of these fish communities, Furthermore, these barriers enhance the effects of natural isolation by distance and the asymmetry of dispersal flows. This may be a slow process, but it can create significant isolation and result in genetic disparity. In particular, populations furthest upstream having low migration rates could be even more subject to genetic impoverishment. This study serves as a first report of its kind for a river system on the Indian subcontinent. The results of this study also emphasize the need for appropriate attention towards the creation of fish passages across the dams and weirs that could help in maintaining biodiversity.

**Electronic supplementary material:**

The online version of this article (doi:10.1186/2193-1801-3-385) contains supplementary material, which is available to authorized users.

## Introduction

India is endowed with diverse aquatic habitats due in part to a unique geological history, highly diverse physiography, a monsoon climate and high biotic diversity. The aquatic habitats include an extensive network of rivers and streams made up of medium and minor river systems (Rao 1975). The majority of these are perennial rivers with large seasonal variation in their flows. In India, over the past 60 years, the landscape of many rivers and streams has also been changed artificially. In response to the growing need of water for agriculture, industrialization and domestic use, many dams and reservoirs are constructed (Khedkar et al. 2014a). Water flow is now highly regulated and is often stored in reservoirs that impound nearly all medium and large rivers. Dams provide benefits in terms of flexibility to use water when it is needed for irrigation, generation of electricity, and other purposes, but have ecological costs as well.

The fragmentation of rivers due to dams or barriers may adversely affect fish populations by diminishing natural habitats required for all life stages and interfering with migration between populations. This in turn may lead to reduced population size, loss of genetic diversity, inbreeding and possible species extinctions. Dams may diminish natural habitats in rivers (Dynesius and Nilsson 1994) and can act as barriers that interfere with migration between populations, even in linked river segments. These barriers may also inhibit recolonization by neighbouring populations when local extinctions occur. River segments altered by dams can also lead to environmental disparities in terms of the temperature regimes, nutrient levels, substrate sizes, organic matter transport, the availability of lotic and lentic habitats, and the overall flow regimes (Ward and Stanford 1983). As a result, aquatic communities may experience altered seasonal movements, loss of genetic diversity, reduced population sizes and inbreeding (Ellstrand and Elam 1993; Lynch et al. 1995; Antunes Antunes et al. 2006). These alterations can lead directly to population extinctions (Fagan 2002).

Because the effects of such artificial alterations may have disproportionately greater impacts on the ability of migratory species to complete their entire life cycle, a number of studies have been directed toward recognizing the effects of river fragmentation due to dams on migratory fish populations (Dunham et al. 1997; Neraas and Spruell 2001; Morita and Yamamoto 2002). In addition, understanding the consequences of fragmentation may also be important for non-migratory species, such as cyprinids or mastacembelids, that have low clearing capacities to overcome the obstacle. Specifically it has been shown that although the entire fish community can be affected by weirs (Miranda et al. 2005; Poulet 2007), cyprinid species often make up a major portion of such river communities (Winfield and Townsend 1991).

Moreover, many cyprinid species are known to have to cover considerable distances for reproduction and feeding (McKeown 1984; Northcote 1984). One can hypothesise that such species would be sensitive to obstacles (Bainbridge 1958; Ovidio and Philippart 2002) and would be at great risk of population decline and loss of local genetic diversity (Knaepkens et al. 2004). However, relatively few studies have explored the impacts of river fragmentation due to dams and weirs on cyprinid populations. Of the studies done, many have been limited to small-scale observations such as the effects of single obstacles or have been assessed on individual-scale processes such as survival and ⁄ or migration only (Lucas and Frear 1997; Ovidio and Philippart 2002).

In this study we used a broad scale approach to analyze the impact of fragmentation of a river due to a combination of factors including dams and natural barriers on populations of a cyprinid fish, *Catla catla*, and a mastacembelid fish, *Mastacembelus armatus*.

*C. catla* was selected as a model since it is a cyprinid, and although not considered to be migratory, is known to be able to cover distances of approximately 15-20 km against water current to spawn. *C. catla* is a ubiquitous species, and based on its size (commonly from 5–30 cm), surface dwelling habitat and its swimming ability, it can also be considered to be representative of many other cyprinids inhabiting Indian rivers (Jhingran 1968). Another species, *M. armatus,* was also selected for this study since it is an eel like species with a bottom dwelling habit (Rainboth 1996; Vidthayanon 2002), is very common in Indian rivers and relatively easy to sample. *M. armatus* is not subject to manipulation as a source of material for hatchery based seed production and stocking, factors known to interfere with natural processes (Froese and Binohlan 2000).

We used DNA sequence information from the D-loop region of the mitochondrial DNA to identify markers to differentiate populations (Wilson and Cann 1985; Bremer et al. 1996; Nyakaana et al. 2002; Sato et al. 2004; Khedkar et al. 2013) and to explore how both artificial and natural fragmentation of river habitats could impact the genetic structure of cyprinid and mastacembelid populations in the Narmada river in India. Our specific objectives were to evaluate the extent to which fragmentation could (i) prompt population differentiation at the genetic level, (ii) to identify the gross effects of multiple obstacles on the population’s genetic structure in the upstream–downstream riverine regions; and (iii) to assess the effects of dams and a natural water fall on dispersion.

## Materials and methods

### Study area

**Figure 1:**
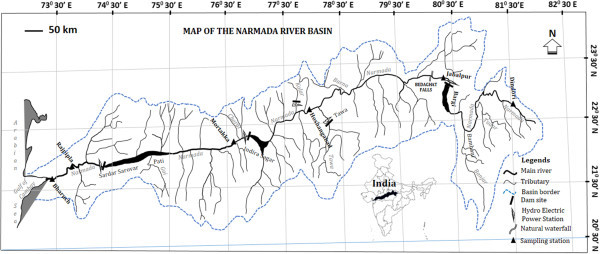
**Map of the Narmada river basin showing location of sampling stations and dams.**

India’s third longest river, known as the Narmada, is 1332 km long and covers a drainage basin totaling approximately 98,796 km^2^. This study considers only the main stretch of the Narmada river and excludes its tributaries. Most of the study sites selected were dominated by cyprinid and mastacembelid fish. The Narmada is fragmented from upstream to downstream by several dams including the Bargi hydroelectric dam (69 m), the Indira Sagar hydroelectric dam (92 m) and Sardar Sarovar hydroelectric dam (136.5 m). In addition a series of six major natural waterfalls in the Bhedaghat area (~30 m height) occur along this part of the river (Table 1, Figure 1). Both the natural and artificial barriers impact the water flow and can potentially limit both upstream and downstream dispersion of the fish.

**Table 1:** **Details of the sampling stations on the Narmada river**

Sampling station	Geographic position			Distance from origin of the river (Km)	Distance from upstream sampling station (Km)	Distance from downstream sampling station (Km)	Presence of barrier up-stream and type	Presence of barrier down-stream and type
	Latitude	Longitude	Elevation (m)					
**Dindori**	22°56’32”N	81°04’34”E	2203	62	–	272	–	Bargi Hydroelectric dam (69 m high).
**Jabalpur**	23°04’55”N	79°54’12”E	1319	334	272	310	Bargi Hydroelectric dam (69 m high).	Series of 6 Natural Waterfalls (30 m high).
**Hoshangabad**	22°45’52”N	77°44’18”E	996	644	310	274	Series of 6 Natural Waterfalls (30 m high).	Indira Sagar Hydroelectric dam (92 m high).
**Mortakka**	22°09’03”N	75°29’54”E	545	918	274	280	Indira Sagar Hydroelectric dam (92 m high).	Sardar Sarovar Hydroelectric dam (136.5 m high).
**Rajpipla**	21°51’48”N	73°30’17”E	113	1198	280	105	Sardar Sarovar Hydroelectric dam (136.5 m high).	–
**Bharuch**	21°42’05”N	73°00’45”E	28	1303	105	–	–	–

### Sampling

Fish were collected from six sampling stations using various nets and gears during April, 2011 - February, 2013 (Figure 1 and Additional file 1: Table S1). Sites were considered for their potential to accommodate cyprinids and mastacembelids (presence of lentic and lotic zones and vegetation shelters) and for their accessibility. Each sampling site was approximately 1500 m long, and all habitats were sampled downstream to upstream until at least 10-12 cyprinid and mastacembelid fish had been caught. We note that *C. catla* fish were not found at Dindori station, and likewise *M. armatus* were not found at Bharuch station. From each specimen a finclip, approximately 1 cm^2^, was obtained and stored in absolute ethanol until laboratory analysis as describe below.

### DNA extraction, PCR and DNA sequencing

Genomic DNA was extracted using the genomic DNA isolation kit (Promega wizard) from a total 43 *C. catla* and 60 *M. armatus* fish. Primers for amplification of the mitchondrial D-loop region of *M. armatus* were designed using the programs PRIMER 3 (Rozen and Skaletsky 2000) and Oligo Calc (Kibbe 2007) by Simgene using a reference sequence obtained from Genbank (EU380216.1). The primers designed for *M. armatus* were: MADF (5′-TTATATGCATTCATTCAGGTACA-3′) and MADR (5′-TAGGGCCCATTTTAACATCT-3′). For *C. catla* the Carp-Pro and Carp-Phe primers were as described by Thai et al. (2004). Amplifications were carried out using an initial denaturation step at 95°C for 2 min followed by 35 cycles of 94°C for 30 s, 57°C for 1 min and 72°C for 1 min along with a final extension at 72°C for 5 min. The amplified fragments were processed for cycle sequencing using the BigDye^®^ Terminator v.3.1 Cycle Sequencing Kit (Applied Biosystems, Inc.) followed by cleanup and bi-directional sequencing using an ABI 3130 Genetic analyzer (Applied Biosystems, Inc.).

### Data analysis

#### Sequence alignments and topographic analysis

Sequences were aligned using Codon code aligner v4.0.3 (CodonCode Corporation, Dedham, MA, USA). Within population diversity was estimated by computing haplotype diversity (H) and nucleotide diversity (π) indices using DnaSP v5.10 (Librado and Rozas 2009) and Arlequin v3.5.1.2 (Excoffier and Lischer 2010). Hierarchical relationships among the populations were analyzed using AMOVA and genetic variance was partitioned using Fst. The phylogenetic analysis included finding the best substitution model in Modeltest 2.1.1 (Darriba et al. 2012) using Akaike Information Criterion (AIC) and Baysian Information Criterion (BIC). The haplotype network was computed using Network v4.6.1.1 (Bandelt et al. 1999) where the haplotype pairwise differences were used to determine the number of mutational steps between haplotypes. A statistical test initially developed to analyze selective neutrality of mutations was implemented to test demographic expansion in recent years (de Jong et al. 2011; Ramos-Onsins and Rozas 2002). As shown by de Jong et al. (2011), these tests are designed to distinguish between neutrally evolving sequences in mutation drift equilibrium and sequences evolving under non-neutral processes including directional and balancing selection and demographic expansion or population contraction. These tests were performed in Arlequin v3.1 using 1000 simulations under a selective model of neutrality. For Mantel test, isolation by distance model was analyzed following the method of Jensen et al. (2005).

## Results

### Diversity indices

Mitochondrial DNA control region (D loop) sequences of 800 bp were obtained and aligned for the individuals from the Narmada river from to two fish species, *M. armatus* and *C. catla* (NCBI Accession numbers KF468051 to KF468110). The sequences of *M. armatus* (n = 60) showed 39 polymorphic sites, accounting for almost 4% of total number of sites examined. Among the polymorphic sites, 7 were singleton variable sites and 2 were parsimony informative sites. Similarly among the individuals representing the *C. catla* population (n = 42), there were 15 variable sites (~2%) including 6 singletons and one parsimony informative site.

### Genetic diversity

**Figure 2:**
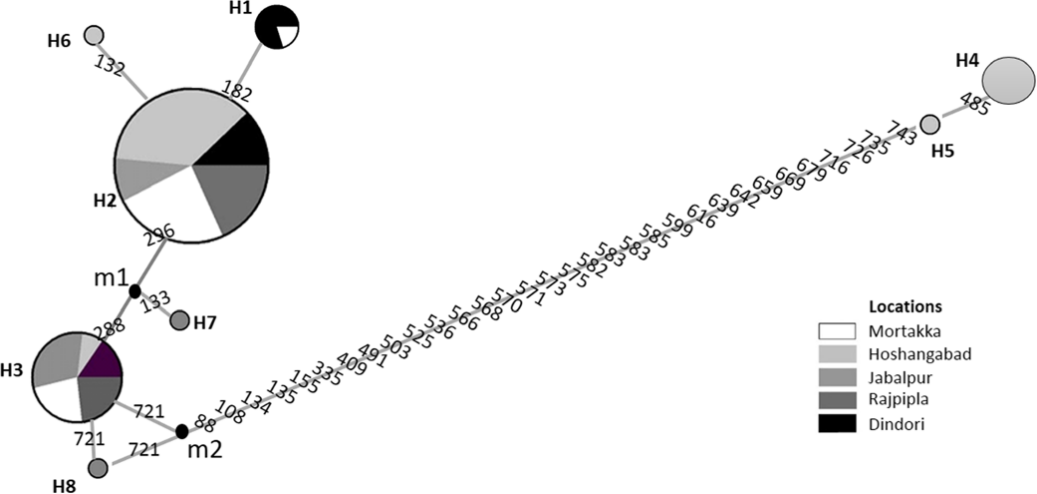
**Median joining network map of haplotypes of** ***M. armatus.***

**Figure 3:**
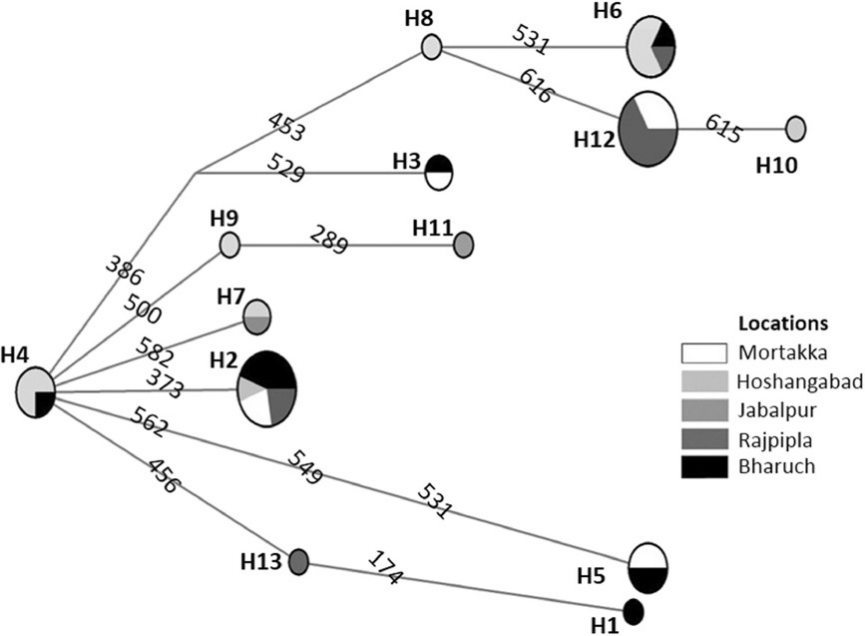
**Median joining network map of haplotypes of** ***C. catla.***

**Figure 4:**
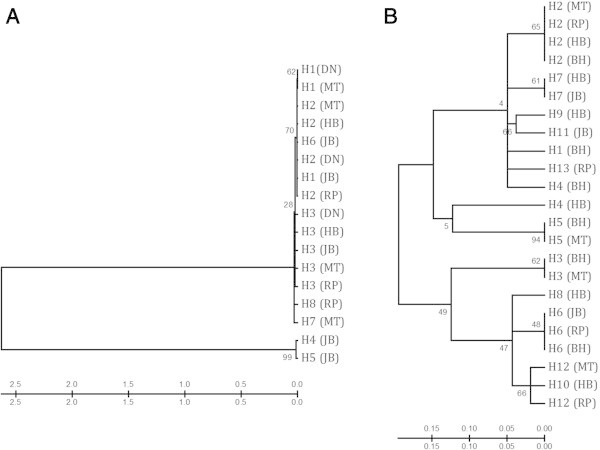
**Haplotype based NJ tree (A)** ***M. armatus*** **; (B)** ***C. catla.***

Eight haplotypes were found in the *M. armatus* populations and thirteen were found in the *C. catla* populations. The distribution and frequency of the haplotypes in each population is shown in Table 2. Haplotypes 2 and 3 are widely distributed in all populations of *M. armatus.* Five unique haplotypes, represented by only single individuals from Jabalpur, Mortakka and Rajpipla populations and three others were found in more than one population (Figure 2). In the *C. catla* populations, haplotype 2 is widely distributed whereas six unique haplotypes represented by only one population and seven others were admixtures of more than one population. Three were found to occur only in the Hoshangabad population (Table 2; Figure 3). Neighbor-joining (NJ) trees showing haplotype relationships were constructed based on the Kimura 2-parameter model, and bootstrap values based on 1,000 replicates shown (Figure 4A and B).

**Table 2:** **Distribution and frequency of different D-loop haplotypes of** ***M. armatus*** **and** ***C. catla*** **in different populations**

Haplotype	Dindori	Jabalpur	Hosangabad	Mortakka	Rajpipla	Bharuch
*Mastacembelus armatus*						
**Hap1**	4 **(** ***0.40)***	0	0	1 ***(0.076)***	0	–
**Hap2**	4 ***(0.40)***	3 ***(0.214)***	12 ***(0.923)***	8 ***(0.615)***	6 ***(0.6)***	–
**Hap3**	2 ***(0.20)***	4 ***(0.286)***	1 ***(0.076)***	3 **(0.231)**	3 ***(0.3)***	–
**Hap4**	0	5 ***(0.357)***	0	0	0	–
**Hap5**	0	1 **(0.071)**	0	0	0	–
**Hap6**	0	1 **(0.071)**	0	0	0	–
**Hap7**	0	0	0	1 ***(0.076)***	0	–
**Hap8**	0	0	0	0	1 ***(0.1)***	–
*Catla catla*						
**Hap1**	–	0	0	0	0	1 ***(0.10)***
**Hap2**	–	0	1 ***(0.125)***	2 ***(0.25)***	2 ***(0.20)***	4 ***(0.40)***
**Hap3**	–	0	0	1 ***(0.125)***	0	1 ***(0.10)***
**Hap4**	–	0	3 ***(0.375)***	0	0	1 ***(0.10)***
**Hap5**	–	0	0	2 ***(0.25)***	0	2 ***(0.20)***
**Hap6**	–	4 ***(0.667)***	0	0	1 ***(0.10)***	1 ***(0.10)***
**Hap7**	–	1 ***(0.167)***	1 ***(0.125)***	0	0	0
**Hap8**	–	0	1 ***(0.125)***	0	0	0
**Hap9**	–	0	1 ***(0.125)***	0	0	0
**Hap10**	–	0	1 ***(0.125)***	0	0	0
**Hap11**	–	1 ***(0.167)***	0	0	0	0
**Hap12**	–	0	0	3 ***(0.375)***	6 ***(0.60)***	0
**Hap13**	–	0	0	0	1 ***(0.10)***	0

Italicized Values in bracket shows the relative haplotype frequency.

For *M. armatus*, the haplotype diversity and nucleotide diversity values for the Jabalpur population were highest (0.791 and 0.0240 respectively) whereas these values were lowest (0.154 and 0.0003 respectively) in the Hoshangabad population (Table 3)*.* In *C. catla,* for the population from Hoshangabad the haplotype diversity was also highest (0.8929), but for this species the nucleotide diversity was highest (0.2404) in the population from Mortakka (Table 3).

**Table 3:** **D-loop sequence divergence values for** ***M. armatus*** **and** ***C. catla*** **for populations from the Narmada river**

Fish populations	Parameters	Sampling stations					
		Dindori	Jabalpur	Hoshangabad	Mortakka	Rajpipla	Bharuch
***Mastacembelus armatus***	No. of Samples (n)	10	14	13	13	10	-
	No. of Polymorphic sites (PS)	3	39	2	4	3	-
	No. of Haplotypes (k)	3	5	2	4	3	-
	Haplotype diversity (H)	0.711 ± 0.0860	0.791 ± 0.0673	0.154 ± 0.1261	0.603 ± 0.1306	0.600 ± 0.1305	-
	Nucleotide diversity (π)	0.0015 ± 0.0011	0.0240 ± 0.0127	0.0003 ± 0.0004	0.0014 ± 0.0010	0.0015 ± 0.0011	-
***Catla catla***	No. of Samples (n)	–	6	8	8	10	10
	No. of Polymorphic sites (PS)	–	6	7	8	6	10
	No. of Haplotypes (k)	–	3	6	4	4	6
	Haplotype diversity (H)	–	0.6000 ± 0.2152	0.8929 ± 0.1113	0.8214 ± 0.1007	0.6444 ± 0.1518	0.8444 ± 0.1029
	Nucleotide diversity (π)	–	0.17333 ± 0.1241	0.1404 ± 0.0994	0.2404 ± 0.1551	0.1481 ± 0.1007	0.2044 ± 0.13120

### Fst analysis

Pairwise comparisons of the genetic variation contained in subpopulations relative to the total populations (Fst) of *M. armatus* were significant when the Jabalpur population was compared with all of the others sampled here. In addition, the comparison of the Dindori and Hoshangbad populations was also significant (Table 4). For the C*. catla* populations, these values are significant only for the comparisons of the Rajpipla and Bharuch populations and Jabalpur and Rajpipla populations (Table 5).

**Table 4:** **Population pairwise Fst comparisons for** ***M. armatus*** **(below the diagonal)**

	Dindori	Jabalpur	Hoshangabad	Mortakka	Rajpipla
**Dindori**	**--**	0.00901*	0.02703*	*0.45946*	*0.22523*
**Jabalpur**	**0.33467**	**--**	0.00000*	0.00000*	0.03604*
**Hoshangabad**	**0.15246**	**0.38583**	**--**	*0.32432*	*0.08108*
**Mortakka**	**0.01433**	**0.35912**	**0.04264**	**--**	*0.62162*
**Rajpipla**	**0.09018**	**0.31342**	**0.17758**	**-0.04100**	**--**

*significant p values (<0.05).

**Table 5:** **Population pairwise Fst comparisons for** ***C. catla (below the diagonal)***

	Jabalpur	Hoshangabad	Mortakka	Rajpipla	Bharuch
**Jabalpur**	**--**	*0.05405*	*0.18018*	*0.04505* *	*0.06306*
**Hoshangabad**	**0.18719**	**--**	*0.38739*	*0.07207*	*0.29730*
**Mortakka**	**0.07238**	**0.01690**	**--**	*0.38739*	*0.34234*
**Rajpipla**	**0.17569**	**0.17440**	**0.01864**	**--**	*0.00901* *
**Bharuch**	**0.18310**	**0.02768**	**-0.00143**	**0.25298**	**--**

*significant p values (<0.05).

For *M. armatus*, the low pairwise Fst value were noted (-0.0410) between Mortakka and Rajpipla may indicate that these populations undergo genetic exchange events. Among the *C. catla* populations, the low Fst values for the Mortakka and Bharuch comparison (-0.00143) may also suggest high levels of genetic exchange (Tables 4 and 5). Also, based on the AMOVA analysis, the majority of the variation was found to be within populations for both species (Table 6).

**Table 6:** **Analysis of molecular variance (AMOVA) among and within populations**

Source of variation	Among populations of ***M. armatus***	Within ***M. armatus***populations	Total	Fst	Among populations of ***C. catla***	Within ***C. catla***populations	Total	Fst
**d.f.**	**4**	**55**	**59**	0.3833*	**4**	**37**	**41**	0.114**
**Sum of squares**	**76.321**	**148.212**	**224.533**		**11.295**	**50.300**	**61.595**	
**Variance component**	**1.372**Va	**2.694**Vb	**4.066**		**0.175**Va	**1.359**Vb	**1.359**	
**Percentage of variation**	**33.74**	**66.26**	**--**		**11.45**	**88.55**	**--**	

Va-Variation among groups; Vb-Variation among populations within groups.

*significant p values (<0.05), **significant p values (<0.01).

To further analyze the potential for gene flow among populations at different locations, we conducted an analysis in ARLEQUIN to obtain M values (Tables 7 and 8). A value greater than two here indicates gene flow between populations (Mallet 2001; Hebert et al. 2004). More than half of our M values are greater than two, and for each of the species, one of the M values is infinite.

**Table 7:** **Matrix of M values for** ***M. armatus***

	Dindori	Jabalpur	Hoshangabad	Mortakka	Rajpipla
**Dindori**	**--**				
**Jabalpur**	**0.99402**	**--**			
**Hoshangabad**	**2.77964**	**0.79590**	–		
**Mortakka**	**34.40182**	**0.89230**	**11.22727**	**--**	
**Rajpipla**	**5.04464**	**1.09529**	**2.31561**	**infinite**	**--**

**Table 8:** **Matrix of M values for** ***C. catla***

	Jabalpur	Hoshangabad	Mortakka	Rajpipla	Bharuch
**Jabalpur**	**--**				
**Hoshangabad**	**2.17115**	**--**			
**Mortakka**	**6.40838**	**29.09091**	**--**		
**Rajpipla**	**2.34597**	**2.36696**	**26.32727**	**--**	
**Bharuch**	**2.23077**	**17.56350**	**infinite**	**1.47643**	**--**

**Figure 5:**
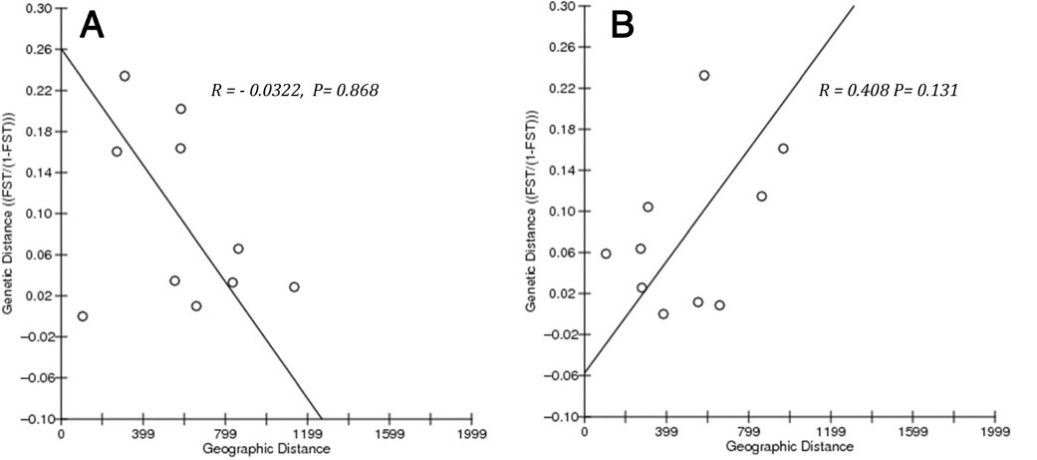
**Scatter plot of genetic vs. geographic variation with RMA regression (A)** ***M. armatus;*** **(B)** ***C. catla.***

Non-significant correlations for isolation by distance were noticed in *M. armatus* populations (Mantel Test; Z = 472.20, r = -0.0322, one sided P = 0.868 from 10,000 randomizations). RMA regression analysis also revealed a negative isolation-by-distance relationship in case of *M. armatus* (y = 0.2607x -2.837, r^2^ = 0.104) (Figure 5A). For the *C. catla* populations there was a positive correlation for isolation by distance (Mantel test; Z = 466.05, r = 0.408, one sided P = 0.1312 from 10000 randomizations) (Figure 5B).

There was significant support for the model of sudden expansion of population size as well as spatial expansion for the *M. armatus* population at Jabalpur (SSD = 0.7924, p = 0.000 and SSD = 0.129, p = 0.0400 respectively). In *C. catla* there is no significant support for either sudden expansion or spatial expansion in any of the populations. The values for Harpending’s raggedness index were non-significant in all populations belonging to both species (Table 9).

**Table 9:** **Analysis of populations to deduce demographic and spatial expansion events**

Statistics	Dindori	Jabalpur	Hoshangabad	Mortakka	Rajpipla	Bharuch
***M. armatus*** **Demographic expansion**						
SSD*	0.05702	0.79242	0.03195	0.10070	0.08515	–
p- values	0.08000	0.00000	0.11000	0.10000	0.21000	**--**
Raggedness index	0.06765	0.37640	0.76331	0.36736	0.31111	**--**
p- values	0.77000	0.95000	0.66000	0.10000	0.24000	**--**
**Spatial expansion**						
SSD*	0.00738	0.12927	0.01297	0.07936	0.05586	**--**
p- values	0.61000	0.04000	0.20000	0.14000	0.21000	**--**
***C. catla*** **Demographic expansion**						
SSD*	–	0.14614	0.01264	0.08586	0.19467	0.08154
p-values	–	0.10000	0.70000	0.04000	0.06000	0.07000
Raggedness index	–	0.27556	0.08291	0.24362	0.62519	0.19605
p- values	–	0.33000	0.73000	0.12000	0.05000	0.08000
**Spatial expansion**						
SSD*	–	0.05272	0.01264	0.06637	0.13729	0.07079
p- values	–	0.50000	0.67000	0.34000	0.09000	0.27000

(*SSD-sum of squared deviations).

## Discussion

Concerns over habitat alterations and the subsequent effects on biodiversity have received much scientific attention during last few decades. Particularly for aquatic diversity, conservationists are concerned that dams may fragment and diminish natural habitats in rivers (Dynesius and Nilsson 1994; Khedkar et al. 2014a) and create environmental disparities (Ward and Stanford 1983) that can result in the loss of genetic diversity, reduced population sizes and inbreeding (Ellstrand and Elam 1993; Lynch et al*.*^1995^). Ultimately, this may lead to population extinctions (Fagan 2002). Evaluation of Indian rivers for the possible effects of fragmentation of river habitats has not been adequately addressed, and in part this study is first effort of its kind to evaluate the possible effects of fragmentation of the Narmada river of India using genetic approaches. Prior to this study, no records of genetic studies for the Narmada river fish have been found (Khedkar et al. 2014b).

The results of our study indicate several things. First, using the data from mtDNA control region (D loop), overall levels of genetic variation of *M. armatus* are moderate as compared to other teleostean fishes (Panarari-Antunes et al. 2012; Librado and Rozas 2009; Aboim et al. 2005; Stefanni and Thorley 2003; Salzburger et al. 2003; Lee et al. 1995; Fajen and Breden 1992). In terms of populations, the *M. armatus* population from Jabalpur is naturally isolated due to a waterfall fall on downstream side and a dam on the upstream side. This population had high levels of nucleotide diversity (0.0240), with up to 39 nucleotide substitutions in some cases. This suggests that this population is evolving independently and may become further genetically differentiated. Similar observations were made for populations of *P. squamosissimus* species from the Parniba and Tocantinus river basins in China (Panarari-Antunes et al. 2012).

In contrast, genetic differentiation among the *C. catla* populations in the Narmada river basin appears to be weaker as overall levels of genetic diversity were only moderate to high. Most of the dams constructed across the river basin are almost 30 years old, and the Bedaghat fall is a natural barrier that is much older. The MtDNA data described here uphold the common assumption that analyzed populations of two native fish species existed in this river before the construction of the dams.

From samples across the entire river, we found eight haplotypes among 60 individuals of *M. armatus* vs. thirteen haplotypes among 42 individuals of *C. catla*. The range of haplotype diversity values (H = 0.154 to 0.791 and 0.6000 to 0.8929, respectively for these two species) and nucleotide diversity values (π = 0.0003 to 0.0240 and 0.1404 to 0.2044 respectively) (Table 3) were similar to values seen in studies by Chen et al. (2006) on Kessle fishes (H = 0.9992, π = 0.0082), Anthunes et al. (2012) on *P. squamonsissmus* (H = 0.690, π = 0.0236).

Genetic data presented here suggest the possibility that *M. armatus* populations, which are invasive to the Narmada basin, originated from Jabalpur. This region was naturally isolated from the rest of downstream regions by the Bedaghat falls a few million years ago, and this would naturally limit genetic exchange for this population on the upstream side. The Jabalpur population has relatively moderate genetic and haplotype diversity, and this may also suggest a recent population expansion after a founder event (Shaw et al. 1992; Carvalho et al. 1996). Comparisons of populations at localities serving as drainages, however, could also provide additional resolution for assessing the levels of genetic differentiation of *M. armatus* populations.

The data revealed different levels of genetic differentiation among the different sampling locations across the basin of the Narmada, Haplotypes 2 and 3 may be treated as ancient haplotypes because they are distributed among all populations, whereas the few private haplotypes may be more recently evolved (Figure 2). The genetic diversity within *C. catla* populations documented here strongly suggests that these fishes were likely derived from different sources, possibly through human translocations, since a mixed haplotype distribution was observed (Figure 3). No significant genetic divergences were revealed in *C. catla*. This may be explained by high rates of gene flow between populations of this species within this system*.*

*C. catla* is also widely used for aquaculture in India because of its simple culture needs and defined seed production technologies (Jhingran 1968). Many hatcheries are located in the Narmada basin, particularly at the Bharuch region, and artificial seed products may be reentering the river channel further impacting genetic diversity. Also from the median joining networks, a higher degree of haplotype sharing between localities was seen for *M. armatus* as compared to the *C. catla* populations (Figures 2 and 3). Therefore, the patterns of genetic diversity seen within *C. catla* may also reflect both inbreeding and genetic drift type events (Nei et al. 1975; Wishard et al. 1984).

For *M. armatus*, the pairwise Fst values indicate some significant amounts of genetic exchange between the Mortakka and Rajpipla populations in spite of the artificial barrier created by the Sardar Sarowar dam. This suggests that these locations may be connected through a sub tributary network that allows migration. On the other hand, the significantly lower amounts of genetic exchange between the Jabalpur and Dindori populations supports the idea that the presence of the Barghi dam between them promotes fragmentation. Also overall, the pairwise Fst values for the Jabalpur population of *M. armatus* compared to the other populations also indicates significant isolation and supports the role of various barriers in promoting population isolation (Table 4). In the case of *C. catla*, the Fst pairwise comparisons suggest that between some populations, such as the Bharuch and Rajpipla populations, significant amounts of genetic exchange can occur. Here, there is no barrier separating these locations.

The potential for isolation is also considered in the analysis of M values. Here, values of M below 2 imply isolation in the population structure (Mallet 2001). The pairwise comparisons of M values for the *M. armatus* population from Jabalpur with Dindori, Hoshangabad, Mortakka and Rajpipla are all less than this critical value, and this supports the role of dams in promoting genetic fragmentation and population structuring (Table 7). The populations of *C. catla* do not show such structuring, except for the pairwise comparison of M values between the Bharuch and Rajpipla population (M < 2). This result is consistent with the results based on pairwise Fst values as described in the previous section.

The results of demographic analysis by mismatch distribution show evidence of population expansion. This inference is supported, except perhaps for the Jabalpur population of *M. armatus* (Table 9). We can assume that a recent population expansion might have been affected by genetic drift due to a bottleneck or a founder effect in case of *M. armatus*. The demographic and spatial expansion values for *M. armatus* are significant for Jabalpur population only (Table 9).

### Isolation by distance

Our sampling was restricted to the main channel of the Narmada River, and the sites are lined up in accordance with the stepping stone model of Kimura (1953). According to this model (Kimura and Weiss 1964), in the idealized case the genetic distance between samples increases simply as geographical distance increases. This model also predicts that in the one-dimensional case, the genetic correlation should fall off exponentially with distance. In the case of *M. armatus*, our observations are not in accordance with this predicted pattern because here adjacent populations are significantly differentiated. This is consistent with the notion that other factors, such as the barriers separating these populations, are promoting fragmentation. In contrast, the relationships of the *C. catla* population follow the model pattern in general and do not appear to be so fragmented.

### Upstream-downstream structure

The variation in genetic diversity along the upstream-downstream gradient is another parameter potentially influenced by the presence of dams. We observed that haplotype and nucleotide diversities are not lined up in a manner consistent with a simple upstream-downstream gradient in populations of both fish species. However, haplotype diversity may be sensitive to sampling bias in that only about 10 individuals per population collected at each sampling site. Additional sampling may reveal the presence of undetected haplotypes.

A number of factors may explain the genetic diversity values seen for the *M. armatus* populations. First, the upper part of the Narmada river studied here is characterized by a natural waterfall and a narrow channel, and it is known that fish population sizes are often correlated to the amount of available habitat (Frankham 1996; Hanfling et al. 2002). Also, *M. armatus* may be subject to a range-edge effect (Arnaud-Haond et al. 2006) since it was more difficult to catch enough specimens in the sites furthest upstream. No individuals were caught at the last locality (Bharuch). Specifically for *M. armatus*, the nature of the dispersion between sampling points, due to the river flow and the obstacles, also suggests that the downstream sites are not receiving new haplotypes from upstream localities.

In the case of *C. catla*, this same dispersion process may maintain a high level of genetic diversity downstream, while genetic drift is less influential in the upstream sampling sites (Lacy 1987). Combining these observations, it can be hypothesized that the *M. armatus* populations within the Narmada river are organized as a single metapopulation composed of local populations exchanging individuals by migration processes (Levins 1970; Hanski and Gilpin 1991). Therefore in this case, the applicability of the stepping-stone model can be rejected.

Dams and or waterfalls, which are designed to prevent upstream movements, seem here in some cases to be sufficient obstacles to induce detectable genetic consequences. In other studies of small species such as bullhead, it has been shown that obstacles only about 20 cm high can prevent upstream movement. Individuals of species such as *Cottus gobio* (Utzinger et al. 1998) and barbel (*Barbus barbus*) individuals are stopped or slowed in their upstream dispersion by a barrier 40 cm high weir (Lucas and Frear 1997). This study confirms that in some cases both dams and water falls can contribute to the asymmetry of dispersion and a decrease in genetic diversity of fish populations.

### Data set quality and potential bias

#### The sampling scheme

Our sampling strategy was to survey as many sites as possible between obstacles that may impact the river habitat structure. The main objective of this study was to assess the extent to which genetic differentiation of populations can be related to fragmentation. The Narmada river system was chosen because there has been extensive fragmentation due to the presence of waterfalls and dams.

In artificially fragmented systems, the number of obstacles is strongly correlated with water way distances (Meldgaard et al. 2003). Dams are generally positioned such that the distance between two consecutive obstacles is about 250 km on average. This holds true for the Narmada system, except that there is also a natural waterfall within 60 kms of the Barghi dam. Due to this spatial configuration, here the effects of waterway distance and the number of obstacles were studied independently.

Lastly, only sampling points of the main Narmada River were considered here. Tributaries are known to have influences on communities inhabiting the main stream of drainage basins, and could also be involved in the demographic and genetic structuring of the Narmada populations (Hitt and Angermeier 2006; Khedkar et al. 2014a). However, for some species, Carlsson et al. (1999) suggested that tributaries may represent ecologically differentiated and locally adapted populations that only rarely disperse in to the main stream, and vice versa. Thus, consideration of only the main Narmada river may be an efficient way to study the genetic structure of these fish populations, although it would be useful to investigate in future studies. the role potentially played by the tributaries.

## Conclusions

The results reported herein for *M. armatus* and *C. catla* populations from the Narmada river clearly show evidence, in some cases, for fragmentation effects by dams and a water fall, and that these barriers contribute to the genetic isolation and differentiation of fish populations. The prevention of movements, either completely from downstream to upstream, or partially from upstream to downstream, by dams or other barriers, enhance the natural effects of isolation by distance and the asymmetry of the dispersal flows. Consequently, populations, especially those furthest upstream, would be expected to have very low immigration rates and be more subject to genetic impoverishment. This study can also provide methodological guidance for future studies of such ecological situations. Sampling a long river segment such as the Narmada with multiple obstacles is certainly a more efficient way to assess the impact on fish dispersal.

Finally, our study does suffer from some biases. Some sample sizes were low, and in some cases it was not possible directly sample upstream and downstream of each barrier. Additional studies in the future may address these biases by including samples of tributaries and other potential sources of input into these populations.

## Electronic supplementary material

Additional file 1: Table S1: Details of fishing nets used for fish sampling. (DOCX 33 KB)
