# Supplementary material for: Genetic fragmentation in India’s third longest river system, the Narmada
Source: Springerplus. 2014 Jul 28;3:385. doi: 10.1186/2193-1801-3-385 (PMC4130967; doi:10.1186/2193-1801-3-385)
Supplement: Supplementary file 1 — Additional file 1: Table S1: Details of fishing nets used for fish sampling. (DOCX 33 KB) [file 40064_2014_1106_MOESM1_ESM.docx]

**Additional file 1: Table S1.** Details of fishing nets used for fish sampling

| **Sr. No.** | **Net type** | **Mesh size (mm)** | **Dimensions (Meter)** |
| --- | --- | --- | --- |
| 1 | Gill net-1 | 152 | 3X70 |
| 2 | Gill net-2 | 101 | 3X50 |
| 3 | Gill net-3 | 75 | 3X30 |
| 4 | Gill net-4 | 50 | 3X30 |
| 6 | Drag net | 15 | 4X50 |
| 7 | Cast net | 10 | 10 Ø |
